# Associations Between Psychosocial Measures and Digital Media Use Among Transgender Youth: Cross-sectional Study

**DOI:** 10.2196/25801

**Published:** 2021-08-13

**Authors:** Brittany J Allen, Zoe E Stratman, Bradley R Kerr, Qianqian Zhao, Megan A Moreno

**Affiliations:** 1 Department of Pediatrics University of Wisconsin School of Medicine and Public Health University of Wisconsin-Madison Madison, WI United States; 2 Department of Biostatistics and Medical Informatics University of Wisconsin-Madison Madison, WI United States

**Keywords:** transgender person, internet, sex and gender minorities, well-being, adolescent, mobile phone

## Abstract

**Background:**

Transgender, nonbinary, and gender-diverse (TNG) youth encounter barriers to psychosocial wellness and also describe exploring identities and communities on the web. Studies of cisgender youth connect increased digital technology use with lower well-being, parent relationships, and body image scores as well as increased loneliness and fear of missing out (FOMO). However, little is known about the psychosocial factors associated with digital technology use among TNG compared with cisgender youth.

**Objective:**

This study aims to examine the associations between psychosocial measures and digital technology use and its importance for cisgender and TNG youth.

**Methods:**

We surveyed a nationally representative sample of adolescents (aged 13-18 years) about psychosocial wellness and digital technology use. Psychosocial measures included assessment of well-being, parental relationships, body image, loneliness, and FOMO. Digital media use assessments included the short Problematic and Risky Internet Use Screening Scale-3 and the Adolescent Digital Technology Interactions and Importance (ADTI) scale and subscales. We compared psychosocial measures between gender identity groups. We also compared stratified correlations for psychosocial measures (well-being, parent relationships, body image, loneliness, and FOMO) with ADTI and Problematic and Risky Internet Use Screening Scale-3 scores between gender identity groups. All comparisons were adjusted for age, race, and ethnicity.

**Results:**

Among 4575 adolescents, 53 (1.16%) self-identified as TNG youth. TNG youth had lower scores for well-being (23.76 vs 26.47; *P*<.001), parent relationships (19.29 vs 23.32; *P*<.001), and body image (13.50 vs 17.12; *P*<.001), and higher scores for loneliness (9.28 vs 6.55; *P*<.001) and FOMO (27.93 vs 23.89; *P=*.004), compared with cisgender peers. In a pattern different from that of their cisgender peers, better well-being scores and body image for TNG youth predicted higher problematic internet use (PIU) scores (correlation coefficients of 0.32 vs −0.07; *P*=.004 and 0.26 vs −0.21; *P*=.002, respectively). FOMO was a stronger positive predictor of higher ADTI total and subscale scores for cisgender youth compared with TNG youth.

**Conclusions:**

Overall, this study supports previously demonstrated disparities in the psychosocial wellness of TNG youth and adds that these disparities include loneliness and FOMO. This study shows prediction of PIU by both higher well-being and better body image, indicating that PIU may not be unilaterally driven by problematic factors among TNG youth. We suggest that this may be because of the specific digital media functions that TNG youth engage with as a disenfranchised population.

## Introduction

### Background

Transgender, nonbinary, and gender-diverse (TNG) youth are a marginalized population that experiences multiple barriers to psychosocial wellness. Experiences of discrimination and oppression are thought to lead to minority stress, which can lead to an increased risk of various negative health effects such as psychological distress, eating disorders, and suicidality [1-3]. Despite being a varied population, it is well recognized that TNG adolescents have disparities in different aspects of psychosocial wellness, including happiness [4] and parent support [5]. Although TNG youth may also experience lower body image and overall quality of life, these can improve with gender-affirming therapies [6,7].

Social support, community connectedness, and coping strategies may protect the psychosocial wellness of TNG youth [1]. However, this population also identifies barriers to support and to identity exploration and expression [8,9]. TNG youth may, therefore, compensate for the risk of poor social support in their communities by using the internet and social media to connect with others to access social and informational support they may not receive elsewhere [10]. How factors in psychosocial wellness are related to TNG youth’s digital media use is not well understood.

In general youth populations, several negative psychosocial measures have been associated with high levels of digital media use. Lower well-being and higher rates of suicidal thoughts are associated with high levels of internet use among adolescents [11]. In addition, depression and higher perceived stress scores are associated with problematic internet use (PIU) [12-14], which is defined as use that is *“*risky, excessive, or impulsive” and leads to *“*physical, emotional, functional, or social impairment” [15].

Other areas of disparity in psychosocial wellness for TNG youth, including low parent support, body image, and well-being, have also been tied to media use in general adolescent populations. For example, increased parental control, restrictive mediation, and parental neglect predict smartphone addiction [16]. Good parent-child communication and web-based parent support, such as being friends with one’s parents on Facebook, are associated with decreased PIU [17,18]. The use of social media is also associated with an increase in concern about body image [19], with increased use associated with increased body dissatisfaction across genders [20]. The barriers that TNG youth experience in these psychosocial domains may influence how and the degree to which they use digital media.

Two additional factors connected to social media use that may be areas of vulnerability for TNG youth are loneliness and fear of missing out (FOMO), the tendency to feel anxious over missing out on rewarding experiences of others. These factors have not been studied in TNG youth but have been associated with digital media use in general adolescent populations [21,22], including frequent social media checks [23]. As gender identity minorities, TNG youth may be at risk of increased loneliness and FOMO, which may impact digital media use in this group. Notably, belonging, the opposite of loneliness, has been identified as a mediator of positive outcomes for TNG people. Community belonging fully mediates the relationship between transgender identity and well-being in TNG adults [24]. In TNG youth, school belonging is associated with decreased drug use among TNG youth [25] and better mental health, and it also mediates the relationship between peer victimization and mental health concerns [26]. Understanding disparities in loneliness and FOMO in TNG youth and the intersection of those factors with digital media may help identify opportunities to facilitate belonging in web-based spaces using evidence-based interventions [27].

Although multiple negative psychosocial wellness factors have been associated with digital media use or PIU, more typical patterns of internet use have also been associated with well-being or life satisfaction in some studies. Better overall well-being is associated with the use of social media to connect with others [28]. Similarly, a study in which participants had to decrease their social media use resulted in a decrease in self-reported life satisfaction [29]. Lesbian, gay, bisexual, transgender, queer, and questioning (LGBTQ+) youth, in particular, are more likely to identify web-based friends and to describe them as more supportive than in-person friends [30]. During the COVID-19 pandemic, LGBTQ+ youth identify the specific importance of web-based spaces and support during a time when they may be stuck at home with unsupportive family members [31]. Thus, previous research suggests potential benefits and risks associated with digital media use, especially for LGBTQ+ youth.

### Objectives

Given that TNG youth identify the importance of web-based spaces while simultaneously experiencing risks to psychosocial wellness, an understanding of the relationship between psychosocial measures and digital technology use is critical for cultivating positive digital experiences for this at-risk population. The aim of this study is to examine the associations between psychosocial measures and digital technology use and its importance for cisgender and TNG youth. We hypothesized that TNG youth would show disparities in measures of psychosocial wellness and that PIU and digital media importance would be tied to negative psychosocial measures in both TNG and cisgender youth.

## Methods

### Setting

We conducted a secondary analysis of a cross-sectional survey administered between February and March 2019. This survey was conducted using Qualtrics, a web-based survey platform. The study was approved by the University of Wisconsin-Madison Education and Social Behavioral Sciences Institutional Review Board.

### Participants

We used a survey panel approach to study a representative sample of adolescents in the United States. Panels facilitate increased speed of data collection with a wide geographic reach compared with traditional approaches for survey distribution [32]. Due to this increased efficiency and ability to provide demographic samples within 10% of their reciprocal US population values, Qualtrics was selected as the survey platform [33]. Recruited participants had previously signed up to get survey invitations through Qualtrics. Upon joining Qualtrics, panelists were asked to complete demographic assessments, which allow relevant survey invitations to be targeted to potentially eligible sample populations. Participants received invitations by email, and the survey remained open until the requested number of participants had completed the survey.

Adult parents who were US residents who spoke English, had adolescent children (aged 13-18 years), and had signed up for survey panel participation were recruited by a Qualtrics survey manager. Information was provided to parents who had potentially eligible children and were interested in the study. Consent was obtained from parents of children aged between 13 and 17 years at the beginning of the survey; adolescents who were aged 18 years gave their own consent before beginning the survey. Participants aged 13-17 years also provided web-based assent before survey initiation. Youth participants had the option to discontinue the survey at any time without loss of benefits. Participants completed demographic information as part of this survey (refer to the full survey in Multimedia Appendix 1) and were included in the study if they answered the question that asked them to describe their gender identity. Youth were excluded if they selected *Preferred not to answer* in response to the question about their gender identity. Although some youth that choose *Preferred not to answer* may be gender-questioning, we deemed it inappropriate to make assumptions about whether this group was primarily cisgender, gender-questioning, or TNG.

### Measures

The full text for all survey measures and scoring summaries can be found in Multimedia Appendix 1.

#### Digital Media Use Measures

##### Problematic and Risky Internet Use

The validated short version of the Problematic and Risky Internet Use Screening Scale-3 (PRIUSS-3) was used to measure problematic and risky internet use [34]. This scale includes three questions that evaluate anxiety when away from the internet, loss of motivation when on the internet, and feelings of withdrawal when away from the internet. A Likert scale was used to measure how often participants experienced these items (0=never, 1=rarely, 2=sometimes, 3=often, and 4=very often). A summed score of three or more on PRIUSS-3 indicates that the participant is at risk for PIU. The α coefficient for this scale was .87.

##### Adolescent Digital Technology Interactions and Importance

Perceived motivations for adolescents’ technology interactions were measured using the validated Adolescent Digital Technology Interactions and Importance (ADTI) scale [35]. There are three ADTI subscales: factor 1, technology to bridge web-based and offline experiences and preferences; factor 2, technology to go outside one’s identity or offline environment; and factor 3, technology for social connection. Each of the 18 items included in the ADTI is associated with one of the subscales. For example, participants were asked how important it was to use social media platforms to “Provide an important accomplishment or update on your life using social media” (factor 1), “video chat” (factor 3), “manage my mood” (factor 2), and “create a profile with a different identity” (factor 2). A five-point Likert scale was used to score perceived importance levels (1=not at all important and 5=extremely important). A higher perceived importance of technology use was indicated by a higher summation score. The α coefficient was .95 for the total ADTI scale. The α coefficients for the three subscales were .90 (factor 1), .92 (factor 2), and .89 (factor 3).

#### Psychosocial Wellness Measures

##### Overall Well-being

Well-being was measured using the validated Short Warwick-Edinburg Mental Well-being Scale [36]. This seven-item scale asks participants to specify how often in the past 2 weeks they experienced the following feelings or experiences: “I’ve been feeling useful,” “I’ve been dealing with problems well,” and “I’ve been able to make up my own mind about things.” Participants responded based on a five-point Likert scale (1=none of the time and 5=all of the time). A summary score was calculated by adding the individual responses for each item. A higher summary score indicated higher levels of well-being. The α coefficient for this measure was .91 in the general population [36].

##### Parental Relationships

To assess parental relationships, we used the eight-question Parent-Adolescent Relationship Scale [37]. This scale consisted of three statements about the adolescent’s identification with the parent, such as “I think highly of him/her,” assessed with a Likert scale of 0=strongly disagree to 4=strongly agree. In addition, this scale includes questions about parent-adolescent relationships, such as “How often does she/he praise you for doing well?” or “How often does she/he blame you for her/his problems?” These were scored with responses ranging from 0 to 4 (0=never and 4=always). Three questions (Multimedia Appendix 1) were reverse scored, as they were framed with negative connotations. A summary score was calculated, with a higher numeric output indicating better parental relationships. The α coefficient for this scale was .68.

##### Body Image

Body image was assessed using the previously validated Body Image Scale, which consists of four items: “I would like to change a good deal about my body,” “I am satisfied with my looks,” “I would like to change a good deal about my looks,” and “I am satisfied with my body” [38]. Each is answered based on a six-point Likert scale, ranging from 1 for “does not apply at all” and 6 for “applies exactly.” A summary score was generated by summing all items; items 1 and 3 were reverse scored because of their negative framing. A higher summary score indicates a more positive body image. The α for this four-item scale was .82 [38].

##### Loneliness

Loneliness was measured using subscale questions from the validated Comprehensive Inventory of Thriving [39]. Participants were asked to agree or disagree with the following statements: “I feel lonely,” “I often feel left out,” and “there is no one I feel close to,” using a five-point Likert scale (1=strongly disagree and 5=strongly agree). A summary score for each participant was calculated, with a higher score indicating increased loneliness. The range of α coefficients for this subscale previously published is .79 to .87 [39] and the α coefficient in our study was .90.

##### Fear of Missing Out

FOMO was measured via a 10-item scale that has been used in previous studies [40-42], which includes statements such as “I get worried when I find out my friends are having fun without me” and “It bothers me when I miss an opportunity to meet up with friends” [43]. Response options were based on a Likert scale (1=not at all true of me and 5=extremely true of me). A summary score was computed for each participant by averaging the responses of all 10 statements. The α coefficient for this scale was .87 [43].

##### Demographics

Demographic questions assessed age, gender, race, and ethnicity. Participants were asked to identify their age by selecting a whole number (12-18) in response to the question, “What is your age in years?.” Gender was assessed by asking, “Which response best describes your gender?,” with response options of “Female,” “Male,” “Non-binary gender,” “Female to male transgender,” “Male to female transgender,” “Other,” or “Prefer not to answer.” Participants were considered cisgender if they answered “Female” or “Male” and TNG if they answered “Non-binary gender,” “Female to male transgender,” “Male to female transgender,” or “Other.” To assess ethnicity and race, respectively, participants were asked, “Are you of Hispanic, Latino, or Spanish origin or descent?” and “What would you consider your race?” (refer to Multimedia Appendix 1 for the full text). Due to limited power in the TNG group, racial groups were dichotomized into White or Caucasian people and people of color and ethnicity into non-Hispanic, Latinx, or Spanish people and Hispanic, Latinx, or Spanish people when used as controls.

#### Analysis

Demographic information was compared between gender groups using the Fisher exact test for categorical analysis, which included comparison by age group (13-14 years vs 15-18 years to demonstrate representation of middle school– and high school–aged youth), race, and ethnicity between gender identity groups. A two-tailed *t* test was used to compare age as a continuous variable. Psychosocial outcomes were compared between gender groups while adjusting for age, race, and ethnicity using analysis of covariance in PROC GLM procedure in SAS. Stratified correlations for psychosocial measures (well-being, parent relationships, body image, loneliness, and FOMO) with ADTI and PRIUSS-3 scores were compared between gender identity groups using PROC NLMIXED procedure in SAS while adjusting for age, race, and ethnicity. We compared the regression coefficients of standardized values between the gender groups; in this case, the slopes were equal to the correlation coefficients. All reported *P* values were two-sided, and *P*<*.*05 was used to define statistical significance. Statistical analyses were performed using SAS software (version 9.4; SAS Institute).

## Results

### Demographics

Among 4575 adolescent participants, there were 53 (1.16%) TNG youth. Mean age (cisgender youth: 14.62 years, SD 1.68; TNG youth: 14.57 years, SD 1.66; *P*=.82) and age distribution did not vary between the two gender identity groups. Compared with cisgender peers, TNG youth were less likely to identify their race as White people (26/53, 49% vs 3041/4522, 67.25%; *P*<.001) and more likely to identify their ethnicity as Hispanic (31/52, 60% vs 786/4469, 17.59%, *P*<.001). Few TNG youth identified with transfeminine identities (5/4575, 0.11% of the total study population) compared with youth with nonbinary identities (23/4575, 0.5%) and transmasculine identities (25/4575, 0.55%). Refer to Table 1 for demographic information.

**Table 1:** Demographics of cisgender and transgender, nonbinary, and gender-diverse youth participants (N=4575).

Demographic measure		Cisgender		TNG^a^			*P* value	
		Total	Participant, n (%)	Total	Participant, n (%)			
**Gender identity^b^**								
	Female or feminine identity	4522	2130 (47.1)	53	5 (9.4)	—^c^		
	Male or masculine identity	4522	2392 (52.9)	53	25 (47.2)	—		
	Nonbinary identity	N/A^d^	N/A	53	23 (43.4)	—		
	Total	4575	4522 (98.84)	4575	53 (1.16)	—		
**Age^e^ (years), n (%)**								
	13-14	4506	2160 (47.94)	53	29 (54.72)	.34		
	15-18	4506	2346 (52.06)	53	24 (45.28)	.34		
**Ethnicity, n (%)**								<.001
	Non-Hispanic	4469	3683 (82.41)	52	21 (40.38)			
	Hispanic	4469	786 (17.59)	52	31 (59.62)			
**Race, n (%)**								<.001
	White	4521	3041 (67.25)	53	26 (49.06)			
	Black	4521	692 (15.3)	53	5 (9.43)			
	Native	4521	139 (3.07)	53	12 (22.64)			
	Asian	4521	227 (5.02)	53	8 (15.09)			
	Multiracial	4521	219 (4.84)	53	2 (3.77)			
	Other	4521	204 (4.51)	53	0 (0)			

^a^TNG: transgender, nonbinary, and gender-diverse.

^b^No youth selected “Other” in identifying their gender identity.

^c^Unable to perform comparison and derive *P* value given that there are different numbers of gender subcategories for cisgender and transgender, nonbinary, and gender-diverse youth.

^d^N/A: not applicable.

^e^Cisgender: mean age 14.62 years (SD 1.68); transgender, nonbinary, and gender-diverse: mean age 14.57 years (SD 1.66); *P*=.82.

### Psychosocial Outcomes

TNG youth had lower scores for well-being (23.76 vs 26.47; *P*<.001), parent relationship scores (19.29 vs 23.32; *P*<.001), and body image (13.5 vs 17.12; *P*<.001) and had higher scores for loneliness scores (9.28 vs 6.55; *P*<.001) and FOMO (27.93 vs 23.89; *P*=.004) compared with cisgender youth. Across these categories, this remained significant when TNG youth were compared with cisgender females, cisgender males, and a combined group of cisgender males and females (Table 2).

**Table 2:** Comparison of mean scores of psychosocial measures between cisgender and transgender, nonbinary, and gender-diverse youth^a^.

Psychosocial outcome measure	Cisgender female and TNG^b^ youth				Cisgender male and TNG youth				Cisgender and TNG youth		
	Cisgender female participants, mean score (SE)	TNG participants, mean score (SE)	*P* value	Cisgender male participants, mean score (SE)		TNG participants, mean score (SE)	*P* value	Cisgender male and female participants, mean score (SE)		TNG participants, mean score (SE)	*P* value
Well-being	26.16 (0.13)	23.76 (0.69)	<.001	26.76 (0.12)		23.76 (0.69)	<.001	26.47 (0.10)		23.76 (0.70)	<.001
Parent relationship	23.46 (0.11)	19.29 (0.62)	<.001	23.18 (0.11)		19.29 (0.62)	<.001	23.32 (0.09)		19.29 (0.62)	<.001
Body image	16.96 (0.11)	13.50 (0.61)	<.001	17.27 (0.11)		13.50 (0.61)	<.001	17.12 (0.09)		13.50 (0.61)	<.001
Loneliness	6.44 (0.09)	9.28 (0.50)	<.001	6.65 (0.09)		9.28 (0.50)	<.001	6.55 (0.07)		9.28 (0.50)	<.001
FOMO^c^	23.88 (0.25)	27.93 (1.37)	.004	23.89 (0.24)		27.93 (1.37)	.004	23.89 (0.20)		27.93 (1.37)	.004

^a^Adjusted for age, race, and ethnicity; all the values are significant compared with *P*=.05.

^b^TNG: transgender, nonbinary, and gender-diverse.

^c^FOMO: fear of missing out.

### Digital Media Use Measures and Psychosocial Outcomes

**Figure 1:**
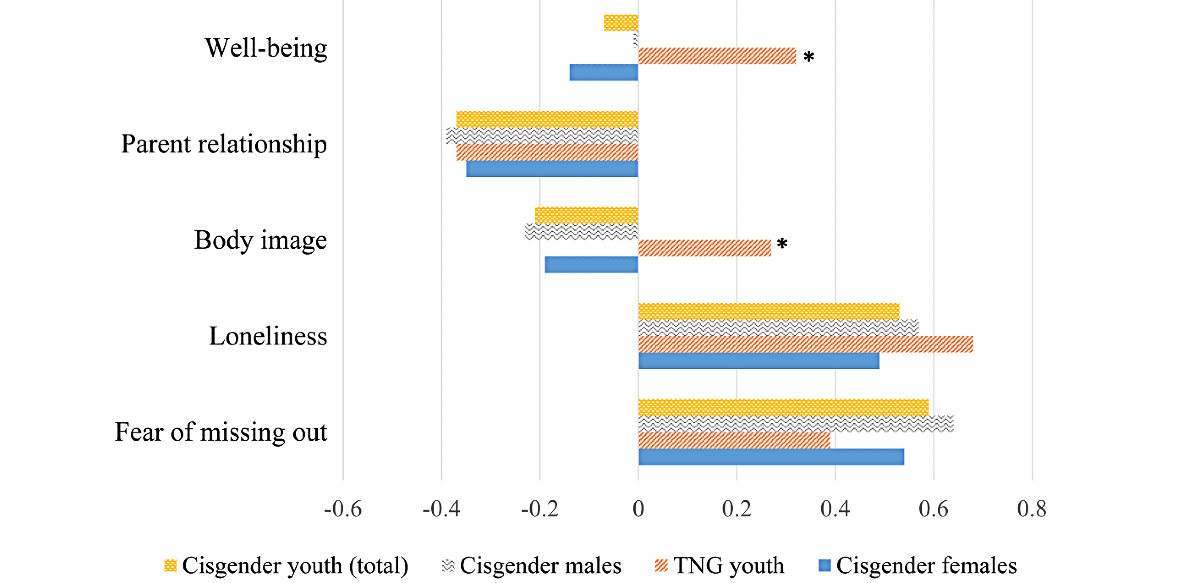
Comparison of correlation coefficients of well-being, parent relationship, body image, loneliness, and fear of missing out versus Problematic and Risky Internet Use Screening Scale-3 for cisgender and transgender, nonbinary, and gender diverse youth. *P<.01. TNG: transgender, nonbinary, and gender-diverse.

When correlations between PRIUSS-3 scores and psychosocial measures were assessed, TNG youth showed patterns that differed from their cisgender peers (Figure 1). Positive body image and higher well-being positively predicted PIU scores for TNG youth (body image: 0.26 and well-being: 0.32), whereas a negative correlation was seen for cisgender youth for both categories (body image: −0.21; *P*=.002 and well-being: −0.07; *P*=.004). TNG and cisgender youth showed similar patterns of PIU correlating negatively with parental relationship scores and positively with loneliness and FOMO. Figure 1 shows the associations between psychosocial outcomes and PRIUSS-3 scores.

There were also some differences in the correlation patterns for cisgender and transgender youth when examining associations between ADTI scores and wellness measures. Better parent relationships predicted higher total ADTI scores in TNG youth (correlation coefficient 0.14), whereas higher parent relationship scores predicted lower total ADTI scores in cisgender males (−0.19; *P*=.04). A significant difference in correlation was not found between TNG youth and cisgender females. Better body image scores also positively predicted higher ADTI-3 scores for TNG youth (correlation coefficient 0.43) compared with slight negative correlation for cisgender youth (−0.01; *P=*.004). Increased FOMO was a positive predictor of ADTI total and subscale scores for youth of all genders, although it was a stronger predictor for cisgender compared with TNG youth. Table 3 shows the associations between the ADTI scores and psychosocial outcomes.

**Table 3:** Comparison of correlation coefficients of well-being, parent relationships, body image, loneliness, and fear of missing out versus digital technology interactions and problematic internet use for transgender, nonbinary, and gender-diverse and cisgender youth^a^.

Outcome and predictor			Cisgender female and TNG^b^ youth							Cisgender male and TNG youth							Cisgender and TNG youth				
			Cisgender females		TNG youth		*P* value		Cisgender males			TNG youth		*P* value		Cisgender males and females			TNG youth		*P* value
**PRIUSS-3^c^ (problematic internet use)**																					
	Well-being	−*0.14*^d^		*0.32*		*<.001*		−*0.01*			*0.32*		*.02*		−*0.07*			*0.32*		*.004*	
	Parent relationship	−0.35		−0.37		.90		−0.39			−0.37		.84		−0.37			−0.37		.96	
	Body image	−*0.19*		*0.27*		*.003*		−*0.23*			*0.27*		*.001*		−*0.21*			*0.26*		*.002*	
	Loneliness	0.49		0.68		.10		0.57			0.68		.38		0.53			0.67		.22	
	FOMO^e^	0.54		0.39		.22		0.64			0.39		.05		0.59			0.39		.11	
**ADTI^f^ total**																					
	Well-being	0.10		0.40		.02		0.23			0.40		.17		0.17			0.40		.06	
	Parent relationship	−0.16		0.14		.06		−*0.19*			*0.14*		*.04*		−0.18			0.13		.05	
	Body image	−0.20		−0.05		.28		−0.29			−0.05		.08		−0.25			−0.05		.15	
	Loneliness	0.28		0.33		.71		0.41			0.33		.53		0.35			0.33		.85	
	FOMO	0.52		0.27		.05		0.67			0.27		.002		0.60			0.27		.01	
**ADTI factor 1 (technology to bridge web-based and offline experiences or preferences)**																					
	Well-being	0.11		0.34		.10		0.23			0.34		.45		0.18			0.34		.23	
	Parent relationship	−0.14		−0.07		.57		−0.23			−0.07		.23		−0.19			−0.07		.36	
	Body image	−0.07		0.15		.17		−0.14			0.15		.06		−0.11			0.14		.10	
	Loneliness	0.19		0.16		.82		0.34			0.16		.23		0.27			0.16		.45	
	FOMO	0.45		0.19		.05		0.61			0.19		.002		0.54			0.19		.01	
**ADTI factor 2 (technology to go outside one’s identity or offline environment)**																					
	Well-being	0.06		0.29		.07		0.18			0.29		.39		0.13			0.29		.20	
	Parent relationship	−0.21		−0.04		.16		−0.34			−0.04		.02		−0.28			−0.04		.05	
	Body image	−0.30		−0.13		.20		−0.30			−0.13		.20		−0.31			−0.13		.19	
	Loneliness	0.32		0.26		.65		0.47			0.26		.11		0.40			0.26		.27	
	FOMO	0.45		0.24		.09		0.65			0.24		<.001		0.56			0.24		.009	
**ADTI factor 3 (technology for social connection)**																					
	Well-being	0.07		0.34		.05		0.21			0.34		.35		0.15			0.34		.15	
	Parent relationship	−0.15		−0.03		.37		−0.19			−0.03		.24		−0.17			−0.03		.29	
	Body image	0.01		0.43		.006		−*0.02*			*0.43*		*.003*		−*0.01*			*0.43*		*.004*	
	Loneliness	0.18		0.35		.22		0.29			0.35		.66		0.23			0.35		.42	
	FOMO	0.44		0.16		.04		0.54			0.16		.006		0.49			0.16		.02	

^a^All comparisons were adjusted for age, race, and ethnicity.

^b^TNG: transgender, nonbinary, and gender-diverse.

^c^PRIUSS-3: Problematic and Risky Internet Use Screening Scale-3.

^d^Values in italics denote statistically significant difference and different patterns of correlation (±) between cisgender and transgender, nonbinary, and gender-diverse youth.

^e^FOMO: fear of missing out.

^f^ADTI: Adolescent Digital Technology Interactions and Importance.

## Discussion

### Principal Findings

This cross-sectional study is the first to explore factors in psychosocial wellness as predictors of PIU and the importance of digital media use among TNG youth. Our findings show continued barriers to psychosocial wellness in TNG youth compared with cisgender youth. In addition, our study shows that the pattern of prediction of psychosocial risk factors with PIU differs in TNG youth, with some positive factors predicting higher PIU scores, suggesting that digital engagement may function differently for this group. Finally, we found that FOMO was a stronger predictor of digital technology importance for cisgender youth compared with TNG youth across ADTI scales and subscales.

The comparison of psychosocial measures for TNG versus cisgender youth is consistent with previous studies that show significant threats to psychosocial wellness in this population [4,5]. Loneliness and FOMO, which have not previously been well studied in TNG youth, were also increased, which may speak to the social isolation that can occur as a gender minority. This is particularly notable given that loneliness and FOMO are constructs inverse to belonging, which has been shown to have a protective mediating effect in TNG youth and adults [24-26]. Future research to better understand the role and development of FOMO in TNG youth may offer insights into and avenues for interventions to facilitate belonging.

Although disparities in psychosocial wellness in TNG youth were consistent across categories, our findings show that positive PRIUSS-3 screens for PIU were predicted by some negative psychosocial experiences in this group (low parent relationship score, loneliness, and FOMO) and also predicted by positive attributes in a pattern different from cisgender peers. Well-being and body image scores predicted positive screens for PRIUSS-3 among TNG youth. In contrast, body image and well-being scores were negatively correlated with positive PRIUSS-3 screens in cisgender peers. This correlation of well-being with the outcome of PIU may complicate the very definition of PIU in this population, as PIU by definition interferes with functions that may be central to well-being. In TNG youth, this finding may represent the complexity of PIU. Although internet and digital media use may be a site of increased web-based bullying for TNG youth [30] and may interfere with day-to-day activities that are considered standard, appropriate activities for youth (such as school), web-based experiences may be varied enough to also support wellness as an alternative to environments where TNG youth may encounter in-person bullying [44] and other forms of harm (again, school).

The prediction of PIU by positive body image for TNG youth may also relate to a function of digital media more specific to this population: the importance of being read as the gender of their identification. TNG youth sometimes experience gender dysphoria (“a marked difference between the individual’s expressed/experienced gender and the gender others would assign him or her” [45]), which may relate to a person’s experience in their body and how their physical appearance is read by others as a certain gender. In general adolescent populations, social media use has been associated with eating disorder behaviors [46]. Limited research suggests that TNG young adults are at higher risk of such behaviors [3], which makes the connection between PIU and positive body image seen here more surprising. This relationship between PIU and body image in TNG youth may be mediated by the digital media function of being able to present and be recognized as their identified gender. Literature from adult transgender populations shows that being able to present (and be *read* by others) as one’s identified gender is related to improved body image [47]. Digital media offers the opportunity to represent oneself using chosen names, pronouns, and selected photographs that may simplify this process compared with offline communities, with different platforms facilitating this in different ways. In addition, disclosure of identity on social media by TNG adults is followed by increased positive sentiment in subsequent posts [48], such that there may be a positive snowball effect from web-based engagement.

This complexity in the relationship of TNG youth with internet use is also evident in the relationship between FOMO and digital media importance. For cisgender youth compared with TNG youth, FOMO is a stronger predictor of higher levels of digital media importance across ADTI and ADTI subscales, measuring overall importance, technology to bridge web-based and offline experiences, technology to go outside one’s identity or offline environment, and technology for social connection. It is notable that, despite increased FOMO in TNG youth, FOMO was both a less powerful predictor of the *importance* of digital media and did not differ as a predictor of PIU for TNG compared with cisgender youth. Although a predominantly cisgender culture in real life reflects cisgender identities as the norm, TNG youth may have increased FOMO as they see only that cisgender narrative in their offline experience, but our results suggest that this feeling of missing out may not be a powerful driver of why this population turns to digital media. This is in line with qualitative descriptions of digital media use, in which TNG youth describe seeking out digital media as a positive resource where they find validation of their identities in their web-based interactions and classify space on the web as a source of informational and emotional support [10,49]. This may show a pivot to digital media as an example of positive action and resilience in TNG youth, as they go on the web to access reflections of their experiences in that of others, a step identified by TNG youth in identity formation [50]. Haimson [51] posits that social media is a *social transition machinery* that facilitates these rites of passage during the process of gender transition. This understanding of web-based interactions driven by motivation for positive interactions (rather than escapism) may help to reframe approaches to building web-based spaces for TNG youth, with increased focus on resilience, belonging through connection, celebration of TNG identities, and community.

### Limitations

Although this study included a large adolescent sample with a prevalence of TNG identity similar to the general US population of approximately 0.5%-3% [52-54], this secondary analysis was not targeted to maximize recruitment of TNG youth, and the absolute number of TNG youth in our study was limited (53/4575, 1.16%). This also limited additional subgroup analyses (eg, by specific gender identity or race and ethnicity). Furthermore, although the ethnic and racial breakdown of participants in this sample mirrors that of the US population [55], a response rate is not available because of the recruitment methods used by Qualtrics. Although these factors may limit generalizability, this study represents an important opportunity to examine the experiences of TNG youth in a large national sample. The TNG youth in this study are more likely to identify as people of color and as Hispanic compared with cisgender youth participants; as such, we controlled for race and ethnicity in our analysis.

In addition, this study did not assess whether TNG youth had accessed gender care. Gender care has been associated with improved body image and quality of life among young TNG people [7], and there is a strong web-based community around gender transition. Indeed, there are a number of YouTube channels, Tumblr blogs, and vlogs [51,56] documenting steps in gender transition, as adolescents and young adults engage in hormone therapy or move through the process of accessing different surgeries. It may be that TNG youth with access to gender care may have improved well-being and body image as well as more web-based engagement in these digital spaces with associated increased measures of PIU. Understanding access to gender care, gender transition, web-based engagement, and PIU would be a rich area for future research.

### Conclusions

Overall, this study is the first to examine the relationship between psychosocial wellness and digital media use as well as the first to show disparities in loneliness and FOMO in TNG compared with cisgender youth. These findings support the importance of a nuanced approach to the interpretation of positive PIU screens in TNG youth. The prediction of PIU by both well-being and improved body image shows that PIU may not be unilaterally problematic among TNG youth, and the definition of and screening tools for PIU may need to be further explored in this population. The pattern of these results may support a picture in which digital media use offers critical functions that may engage and reinforce TNG youth with some strengths in certain areas of psychosocial wellness, including overall well-being and body image. In addition, this highlights the limited role of FOMO in digital media importance in this group compared with cisgender youth, which may offer opportunities to better understand and facilitate resilience and belonging in web-based spaces. In clinical settings, a nuanced, harm reduction approach may assist with counseling and creating a realistic media plan to reduce screen time while honoring that a young TNG person may benefit from specific functions of digital media.

Whether this more complicated picture of PIU applies to other minority populations besides TNG youth will be an important area for future research. A better understanding of positive PRIUSS-3 screens, PIU, and reasons for and predictors of digital media use, particularly in disenfranchised or oppressed populations (such as Black and indigenous youth and other youth of color; lesbian, gay, bisexual, and questioning youth; and disabled youth), will better inform opportunities for intervention and support.

## Appendix

Multimedia Appendix 1 Survey details, with keys to scoring and interpretation.

## Abbreviations

ADTI: Adolescent Digital Technology Interactions and Importance
FOMO: fear of missing out
LGBTQ+: lesbian, gay, bisexual, transgender, queer, and questioning
PIU: problematic internet use
PRIUSS-3: Problematic and Risky Internet Use Screening Scale-3
TNG: transgender, nonbinary, and gender-diverse
