# Supplementary material for: Associations Between Psychosocial Measures and Digital Media Use Among Transgender Youth: Cross-sectional Study
Source: JMIR Pediatr Parent. 2021 Aug 13;4(3):e25801. doi: 10.2196/25801 (PMC8398741; doi:10.2196/25801)
Supplement: Multimedia Appendix 1 [file pediatrics_v4i3e25801_app1.pdf]

Multimedia Appendix 1. Survey Details, with keys to scoring and interpretation.

Teen Initial Demographics

What is your age in years?

1. 12
2. 13
3. 14
4. 15
5. 16
6. 17
7. 18

ADTI (Adolescents Digital Technology Interactions) (1 score)

18 items

3 Factors: Factor 1 (6 items), Factor 2 (7 items), Factor 3 (5 items)

1. Provide an important accomplishment or update on your life using social media
2. Change, add to, or remove from existing content that you or other people have created *(For example: change the text of a status update, remove a photo, add a tag of someone on a photo)*
3. Look into or follow a business or product *(For example: Adidas, Nordstrom)*
4. Plan an event
5. Follow or look into an event you may attend
6. Post a photo that you took for artistic reasons
7. Create a profile with a different identity
8. See what people are up to without asking them about it
9. Direct message, converse, chat, or talk back and forth with another person *(one on one)*
10. Video Chat *(For example: Skype or FaceTime)*
11. Contribute to a private conversation *(For example: messaging or in a private group)*
12. Create a piece of content, such as a text, photo, video or combination of text, photos and videos that will disappear or be impermanent *(For example: a story, Snapchat)*
13. Use a service that allows me to track what I'm doing *(For example: using an app to track your run, steps, heart rate, sleep)*
14. Manage my mood
15. Steal or copy others' identities
16. Use applications or devices that create and transport me to a 3D virtual environments with virtual objects to replace the real everyday-life world. (*For example: using a virtual reality headset such as Oculus or Google Daydream*)
17. Explore my sexuality
18. Build a brand

Responses:

1= Not at all important

2= slightly important
3= Moderately Important
4= Very important
5= extremely important

Summary Score: Yes
Reversed Scored: No

How important, if at all, is it for you to use media platforms for the following purposes?- **Provide an important accomplishment or update on your life using social media**

1. Never
2. Rarely
3. Sometimes
4. Often
5. Very often

How important, if at all, is it for you to use media platforms for the following purposes?- **Change, add to, or remove from existing content that you or other people have created *(For example: change the text of a status update, remove a photo, add a tag of someone on a photo)***

1. Never
2. Rarely
3. Sometimes
4. Often
5. Very often

How important, if at all, is it for you to use media platforms for the following purposes?- **Look into or follow a business or product *(For example: Adidas, Nordstrom)***

1. Never
2. Rarely
3. Sometimes
4. Often
5. Very often

How important, if at all, is it for you to use media platforms for the following purposes?- **Plan an event**

1. Never
2. Rarely
3. Sometimes
4. Often
5. Very often

How important, if at all, is it for you to use media platforms for the following purposes?- **Follow or look into an event you may attend**

1. Never
2. Rarely

3. Sometimes<br>4. Often<br>5. Very often
How important, if at all, is it for you to use media platforms for the following purposes?- **Post a photo that you took for artistic reasons**<br><br>1. Never<br>2. Rarely<br>3. Sometimes<br>4. Often<br>5. Very often
How important, if at all, is it for you to use media platforms for the following purposes?- **Create a profile with a different identity**<br><br>1. Never<br>2. Rarely<br>3. Sometimes<br>4. Often<br>5. Very often
How important, if at all, is it for you to use media platforms for the following purposes?- **See what people are up to without asking them about it**<br><br>1. Never<br>2. Rarely<br>3. Sometimes<br>4. Often<br>5. Very often
How important, if at all, is it for you to use media platforms for the following purposes?- **Direct message, converse, chat, or talk back and forth with another person (one on one)**<br><br>1. Never<br>2. Rarely<br>3. Sometimes<br>4. Often<br>5. Very often
How important, if at all, is it for you to use media platforms for the following purposes?- **Video Chat (For example: Skype or FaceTime)**<br><br>1. Never<br>2. Rarely<br>3. Sometimes<br>4. Often<br>5. Very often
How important, if at all, is it for you to use media platforms for the following purposes?- **Contribute to a private conversation (For example: messaging or in a private group)**

1. Never
2. Rarely
3. Sometimes
4. Often
5. Very often

How important, if at all, is it for you to use media platforms for the following purposes?- **Create a piece of content, such as a text, photo, video or combination of text, photos and videos that will disappear or be impermanent (For example: a story, Snapchat)**

1. Never
2. Rarely
3. Sometimes
4. Often
5. Very often

How important, if at all, is it for you to use media platforms for the following purposes?- **Use a service that allows me to track what I'm doing (For example: using an app to track your run, steps, heart rate, sleep)**

1. Never
2. Rarely
3. Sometimes
4. Often
5. Very often

How important, if at all, is it for you to use media platforms for the following purposes?- **Manage my mood**

1. Never
2. Rarely
3. Sometimes
4. Often
5. Very often

How important, if at all, is it for you to use media platforms for the following purposes?- **Steal or copy others' identities**

1. Never
2. Rarely
3. Sometimes
4. Often
5. Very often

How important, if at all, is it for you to use media platforms for the following purposes?- **Use applications or devices that create and transport me to a 3D virtual environments with virtual objects to replace the real everyday-life world. (For example: using a virtual reality headset such as Oculus or Google Daydream)**

1. Never
2. Rarely
3. Sometimes

4. Often<br>5. Very often
How important, if at all, is it for you to use media platforms for the following purposes?- **Explore my sexuality**<br><br>1. Never<br>2. Rarely<br>3. Sometimes<br>4. Often<br>5. Very often
How important, if at all, is it for you to use media platforms for the following purposes?- **Build a brand**<br><br>1. Never<br>2. Rarely<br>3. Sometimes<br>4. Often<br>5. Very often

**Teen Follow-up Demographics**<br>1. Gender<br>2. Ethnicity<br>3. Race
Which response best describes your gender?<br>1. Female<br>2. Male<br>3. Non-binary gender<br>4. Female to male transgender<br>5. Male to female transgender<br>6. Other<br>7. Prefer not to answer
Are you of Hispanic, Latino or Spanish origin or descent?<br>1. No, not of Hispanic, Latino, or Spanish origin<br>2. Yes, Mexican American, Chicano<br>3. Yes, Puerto Rican<br>4. Yes, Cuban<br>5. Yes, another Hispanic, Latino or Spanish origin<br>6. prefer not to answer
What would you consider your race?<br>1. White/Caucasian<br>2. Black or African American<br>3. American Indian or Alaska Native<br>4. Asian<br>5. Asian Indian<br>6. Other Asian

7. Native Hawaiian/other Pacific Islander
8. Multiracial
9. Other
10. Prefer not to answer
11. Latino/Hispanic/Mexican

PRIUSS-3 (1 score)
3 items

1. How often do you... experience increased social anxiety due to your internet use
2. How often do you... feel withdrawal when away from the internet
3. How often do you... lose motivation to do other things that need to get done because of the internet

Responses:
Never= 0
Rarely = 1
Sometimes = 2
Often = 3
Very often= 4

Summary Score: Yes

- Patients with a total score of greater than or equal to 3 on the PRIUSS-3 are considered at risk

Reversed Scored: No

How often do you... experience increased social anxiety due to your internet use

1. Never
2. Rarely
3. Sometimes
4. Often
5. Very often

How often do you... feel withdrawal when away from the internet

1. Never
2. Rarely
3. Sometimes
4. Often
5. Very often

How often do you... lose motivation to do other things that need to get done because of the internet

1. Never
2. Rarely
3. Sometimes
4. Often
5. Very often

SWEMWBS Well-being (1 Score)
7 items

1. I've been feeling optimistic about the future
2. I've been feeling useful
3. I've been feeling relaxed
4. I've been dealing with problems well
5. I've been thinking clearly
6. I've been feeling close to other people
7. I've been able to make up my own mind about things

Responses:
None of the time= 1
Rarely=2
Some of the time =3
Often=4
All of the time=5
Prefer not to answer=6

Summary Score: Yes
The total score is calculated by summing the individual items. SWEMWBS is scored by transforming the scores according to a raw score to metric score conversion table. A linear transformation of the raw score from SWEMWBS can be used with confidence in parametric analyses, given appropriate distribution. The translation is also only valid when the data are complete, i.e. there are no missing values.

Raw	Metric
7	7.00
8	9.51
9	11.25
10	12.40
11	13.33
12	14.08
13	14.75
14	15.32
15	15.84
16	16.36
17	16.88
18	17.43
19	17.98
20	18.59
21	19.25
22	19.98
23	20.73
24	21.54
25	22.35
26	23.21
27	24.11
28	2503
29	26.02
30	27.03
31	28.13
32	29.31
33	30.70
34	32.55
35	35.00

Reverse Scored: No

Below are some statements about feelings and thoughts. Please check the box that best describes your experience of each over the last 2 weeks. - **I've been feeling optimistic about the future**

1. None of the time
2. Rarely
3. Some of the time
4. Often
5. All of the time
6. Prefer not to answer

Below are some statements about feelings and thoughts. Please check the box that best describes your experience of each over the last 2 weeks. - **I've been feeling useful**

1. None of the time
2. Rarely
3. Some of the time

4. Often
5. All of the time
6. Prefer not to answer

Below are some statements about feelings and thoughts. Please check the box that best describes your experience of each over the last 2 weeks. - **I've been feeling relaxed**

1. None of the time
2. Rarely
3. Some of the time
4. Often
5. All of the time
6. Prefer not to answer

Below are some statements about feelings and thoughts. Please check the box that best describes your experience of each over the last 2 weeks. **- I've been dealing with problems well**

1. None of the time
2. Rarely
3. Some of the time
4. Often
5. All of the time
6. Prefer not to answer

Below are some statements about feelings and thoughts. Please check the box that best describes your experience of each over the last 2 weeks. - **I've been thinking clearly**

1. None of the time
2. Rarely
3. Some of the time
4. Often
5. All of the time
6. Prefer not to answer

Below are some statements about feelings and thoughts. Please check the box that best describes your experience of each over the last 2 weeks. - **I've been feeling close to other people**

1. None of the time
2. Rarely
3. Some of the time
4. Often
5. All of the time
6. Prefer not to answer

Below are some statements about feelings and thoughts. Please check the box that best describes your experience of each over the last 2 weeks. - **I've been able to make up my own mind about things**

1. None of the time
2. Rarely
3. Some of the time
4. Often
5. All of the time
6. Prefer not to answer

Comprehensive Inventory of Thriving: Loneliness(1 score)
3 items

1. I feel lonely
2. I often feel left out
3. There is no one I feel close to

Responses:
Strongly disagree=1
Somewhat disagree= 2
Neither agree nor disagree=3
Somewhat agree=4
Strongly agree= 5

Summary Score: Yes

- Items were negatively phrased such that high score signifies that respondents view themselves as LONELY

Reversed Scored: No

Please agree or disagree with the following statements. - **I feel lonely**

1. Strongly disagree
2. Somewhat disagree
3. Neither agree nor disagree
4. Somewhat agree
5. Strongly Agree

Please agree or disagree with the following statements. - **I often feel left out**

1. Strongly disagree
2. Somewhat disagree
3. Neither agree nor disagree
4. Somewhat agree
5. Strongly Agree

Please agree or disagree with the following statements. - **There is no one I feel close to**

1. Strongly disagree
2. Somewhat disagree
3. Neither agree nor disagree
4. Somewhat agree
5. Strongly Agree

Fear of Missing Out (FOMO) (1 score)
10 item scale

1. I fear others have more rewarding experiences than me.
2. I fear my friends have more rewarding experiences than me.
3. I get worried when I find out my friends are having fun without me
4. I get anxious when I don't know what my friends are up to.
5. It is important that I understand my friends' "in jokes."
6. Sometimes, I wonder if I spend too much time keeping up with what is going on.
7. It bothers me when I miss an opportunity to meet up with friends.

8. When I have a good time it is important for me to share the details online (e.g. updating status).
9. When I miss out on a planned get-together it bothers me.
10. When I go on vacation, I continue to keep tabs on what my friends are doing.

Responses:
Not at all true of me= 1
Slightly true of me= 2
Moderately true of me=3
Very true of me = 4
Extremely true of me =5

Summary Score: Yes
Individual scores can be computed by averaging responses to all ten items and forms a reliable composite measure.

Reversed score: No

Please answer according to what really reflects your experiences rather than what you think your experiences should be. Please treat each item separately from every other item.- **I fear others have more rewarding experiences than me.**

1. Not at all true of me
2. Slightly true of me
3. Moderately true of me
4. Very true of me
5. Extremely true of me

Please answer according to what really reflects your experiences rather than what you think your experiences should be. Please treat each item separately from every other item. - **I fear my friends have more rewarding experiences than me.**

1. Not at all true of me
2. Slightly true of me
3. Moderately true of me
4. Very true of me
5. Extremely true of me

Please answer according to what really reflects your experiences rather than what you think your experiences should be. Please treat each item separately from every other item.- **I get worried when I find out my friends are having fun without me**

1. Not at all true of me
2. Slightly true of me
3. Moderately true of me
4. Very true of me
5. Extremely true of me

Please answer according to what really reflects your experiences rather than what you think your experiences should be. Please treat each item separately from every other item. - **I get anxious when I don't know what my friends are up to.**

1. Not at all true of me
2. Slightly true of me
3. Moderately true of me
4. Very true of me
5. Extremely true of me

Please answer according to what really reflects your experiences rather than what you think your experiences should be. Please treat each item separately from every other item.- **It is important that I understand my friends' "in jokes."**<br>1. Not at all true of me<br>2. Slightly true of me<br>3. Moderately true of me<br>4. Very true of me<br>5. Extremely true of me
Please answer according to what really reflects your experiences rather than what you think your experiences should be. Please treat each item separately from every other item.- **Sometimes, I wonder if I spend too much time keeping up with what is going on.**<br>1. Not at all true of me<br>2. Slightly true of me<br>3. Moderately true of me<br>4. Very true of me<br>5. Extremely true of me
Please answer according to what really reflects your experiences rather than what you think your experiences should be. Please treat each item separately from every other item. - **It bothers me when I miss an opportunity to meet up with friends.**<br>1. Not at all true of me<br>2. Slightly true of me<br>3. Moderately true of me<br>4. Very true of me<br>5. Extremely true of me
Please answer according to what really reflects your experiences rather than what you think your experiences should be. Please treat each item separately from every other item. - **When I have a good time it is important for me to share the details online (e.g. updating status).**<br>1. Not at all true of me<br>2. Slightly true of me<br>3. Moderately true of me<br>4. Very true of me<br>5. Extremely true of me
Please answer according to what really reflects your experiences rather than what you think your experiences should be. Please treat each item separately from every other item. - **When I miss out on a planned get-together it bothers me.**<br>1. Not at all true of me<br>2. Slightly true of me<br>3. Moderately true of me<br>4. Very true of me<br>5. Extremely true of me
Please answer according to what really reflects your experiences rather than what you think your experiences should be. Please treat each item separately from every other item.- **When I go on vacation, I continue to keep tabs on what my friends are doing.**<br>1. Not at all true of me<br>2. Slightly true of me<br>3. Moderately true of me<br>4. Very true of me

5. Extremely true of me

Parent-Adolescent Relationship Scale (1 score)
a) Youth's Identification with the parent(s): 3 items

1. I think highly of him/her
2. She/He is a person I want to be like
3. I really enjoy spending time with him/her

<u>Responses:</u>
Strongly disagree=0
Somewhat Disagree=1
Neither agree or disagree= 2
Somewhat agree=3
Strongly agree= 4

b) Parent-Adolescent Relationship Scale- Parental Supportiveness: 5 items

1. How often does she/he praise you for doing well?
2. How often does she/he criticize you or your ideas? (-)
3. How often does she/he help you do things that are important to you?
4. How often does she/he blame you for her/his problems? (-)
5. How often does she/he make plans with you and cancel for no good reason? (-)

<u>Responses:</u>
Never=0
Sometimes=1
About half the time= 2
Most of the time= 3
Always=4

<u>Summary Score:</u> Yes
Sum values. Possible range is 0 to 32. Value of 24 would be characterized as having a high-quality relationship with his or her parent.

<u>Reverse scored:</u> YES
b) Questions 2,4,5 (Supportiveness)

Please answer the following questions about your relationship with the parent/guardian who took the survey with you today. - **I think highly of him/her**

1. Strongly disagree
2. Somewhat disagree
3. Neither agree nor disagree
4. Somewhat agree
5. Strongly Agree

Please answer the following questions about your relationship with the parent/guardian who took the survey with you today. - **She/He is a person I want to be like**<br>1. Strongly disagree<br>2. Somewhat disagree<br>3. Neither agree nor disagree<br>4. Somewhat agree<br>5. Strongly Agree
Please answer the following questions about your relationship with the parent/guardian who took the survey with you today. - **I really enjoy spending time with him/her**<br>1. Strongly disagree<br>2. Somewhat disagree<br>3. Neither agree nor disagree<br>4. Somewhat agree<br>5. Strongly Agree
Please answer the following questions about your relationship with the parent/guardian who took the survey with you today. - **How often does she/he praise you for doing well?**<br>1. Never<br>2. Sometimes<br>3. About half the time<br>4. Most of the time<br>5. Always
Please answer the following questions about your relationship with the parent/guardian who took the survey with you today. - **How often does she/he criticize you or your ideas?**<br>1. Never<br>2. Sometimes<br>3. About half the time<br>4. Most of the time<br>5. Always
Please answer the following questions about your relationship with the parent/guardian who took the survey with you today. - **How often does she/he help you do things that are important to you?**<br>1. Never<br>2. Sometimes<br>3. About half the time<br>4. Most of the time<br>5. Always
Please answer the following questions about your relationship with the parent/guardian who took the survey with you today. - **How often does she/he blame you for her/his problems?**<br>1. Never<br>2. Sometimes<br>3. About half the time<br>4. Most of the time<br>5. Always
Please answer the following questions about your relationship with the parent/guardian who took the survey with you today. - **How often does she/he make plans with you and cancel for no good reason?**

1. Never
2. Sometimes
3. About half the time
4. Most of the time
5. Always

Body Image Scale (1 score)
4 items
1. I would like to change a good deal about my body. (-)
2. By and large, I am satisfied with my looks.
3. I would like to change a good deal about my looks. (-)
4. By and large, I am satisfied with my body.

Responses:
Does not apply at all= 1
Does not apply well= 2
Applies somewhat = 3
Applies fairly well= 4
Applies well =5
Applies exactly= 6

Summary Score: Yes

Reversed Score: Yes
1,3

Indicate how the following statements applies to you. - **I would like to change a good deal about my body**
1. Does not apply at all
2. Does not apply well
3. Applies somewhat
4. Applies fairly well
5. Applies well
6. Applies exactly

Indicate how the following statements applies to you.- **By and large, I am satisfied with my looks**
1. Does not apply at all
2. Does not apply well
3. Applies somewhat
4. Applies fairly well
5. Applies well
6. Applies exactly

Indicate how the following statements applies to you.- **I would like to change a good deal about my looks**
1. Does not apply at all
2. Does not apply well
3. Applies somewhat
4. Applies fairly well
5. Applies well

6. Applies exactly
Indicate how the following statements applies to you.- **By and large, I am satisfied with my body**<br>1. Does not apply at all<br>2. Does not apply well<br>3. Applies somewhat<br>4. Applies fairly well<br>5. Applies well<br>6. Applies exactly
